# Prevalence and distribution of soil-transmitted helminth infections in India

**DOI:** 10.1186/s12889-017-4113-2

**Published:** 2017-02-16

**Authors:** Nasir Salam, Saud Azam

**Affiliations:** 1 Department of Pharmacology, College of Medicine, Al-Imam Mohammad Ibn Saud Islamic University (IMSIU), Riyadh, Saudi Arabia; 2 Innova-CRO, Riyadh, Saudi Arabia

## Abstract

**Background:**

Understanding the prevalence of soil-transmitted helminth infections is necessary to plan control strategies and focus on highly endemic regions for preventive chemotherapy and improved sanitation facilities. India is known to be endemic for soil-transmitted helminth infections.

**Methods:**

To understand the prevalence, spatial distribution and identify high-risk zones, a systematic search of published literature was carried out based on PRISMA guidelines from the year 2000 to 2015.

**Results:**

A careful screening of the identified literature yielded 39 studies that reported the prevalence of soil-transmitted helminth infections from 19 different states of India. *Ascaris lumbricoides* was the most prevalent parasite. Higher than 50% prevalence was reported from six states. Nearly 90% studies reported the prevalence of more than one parasite species in the same sample population.

**Conclusion:**

This is the first study to comprehensively review the literature associated with soil-transmitted helminth infections from India giving a clear idea of its prevalence, distribution and high endemic areas.

## Background

Soil-transmitted Helminths (STH) infects nearly 2 billion people of world’s population with children being the most affected [1]. According to the World Health Organization (WHO) estimates, 870 million children live in the area of high prevalence. Africa, South Asia and South America are the most affected regions of the world [2]. India alone contributes nearly 25% to the total global cases with 220.6 million children in need of preventive chemotherapy [3]. STH infections rarely cause mortality with diarrhea, abdominal pain and low hemoglobin levels as the immediate outcome of infections, however, the long term effects of these infections are far more sinister as those with infections show reduced cognitive abilities, intellectual capacity and lower work productivity [4]. The warm and moist climate of tropical and subtropical countries provides the ideal environment for the survival of parasite eggs or larvae of these four STH, roundworm (*Ascaris lumbricoides*), whipworm (*Trichuris trichiura*) and hookworm (*Necator americanus, Ancylostoma duodenale*) [5].

The prevalence and control of STH infections is inextricably linked with water quality, sanitation, hygiene practices and socio-economic status in the affected areas [6]. Despite the fact that infection can be cured with either Albendazole or Mebendazole, eradication is difficult, given STH’s feco-oral and penetration-via-skin transmission pattern as the chances of reinfection are very high in population living in affected areas [7]. Control is achieved by targeted use of chemotherapy and improvement of sanitation, drinking water, use of pit-latrines instead of open defecation and good hygiene practices [8–10]. India, with support from WHO, launched a Global Programme for the Elimination of Lymphatic Filariasis in the year 2000 (http://nvbdcp.gov.in/doc/guidelines-filariasis-elimination-india.pdf). Under this programme Diethylcarbamazine (DEC) and Albendazole were administered to people living in filarial endemic areas. In 2015 another nation-wide deworming programme was launched covering nearly 241 million children with STH infection or at risk of developing the infection (http://nrhm.gov.in/nrhm-components/rmnch-a/child-healthimmunization/national-deworming-day-february-2017.html). For such targeted efforts a comprehensive knowledge of the prevalence pattern of these helminth infections needs to be assessed thoroughly. Several studies have been published from India by clinicians and researchers assessing the prevalence and epidemiology of STH infections in different states and municipalities. Such studies have the potential to guide governmental and non-governmental organizations (NGOs) to focus their efforts in highly endemic areas. The purpose of this review is to evaluate the prevalence of STH infections and identify high-risk areas in different regions of India, based on a comprehensive search and analysis of published literature. Such an exercise would help Governmental agencies and NGOs to focus on specific areas of high prevalence for preventive chemotherapy and improved sanitation practices.

## Methods

### Search Strategy and Data extraction

We did a review based on PRISMA (Preferred Reporting Items for Systematic Reviews and Meta-Analyses) guidelines to identify all relevant publications pertaining to the prevalence of STH infections in India. We systematically searched PubMed and Web of knowledge from January 1, 2000 to June 30, 2015. We did not search before the year 2000 because our goal was to inform decision-making rather than providing a historical perspective. In this regard, it is pertinent to mention that STH infections are often a function of sanitation, health care and economic condition. In the last few decades India has seen considerable improvement in sanitation with a reduction of 31% in open defection. Community based and school based mass deworming programs have been undertaken to reduce the burden of STH infections.

We used the following search terms anywhere in the articles: “soil transmitted helminth” or “ascaris” or “trichuris” or “whipworm” or “necator” or “ancylostoma” or “hookworm” AND “India”. We searched without any bar on language or nature of studies. To identify additional studies, reference lists of publications were carefully screened. Initial assessment was based on review of title and abstract of all the studies. Full text of potentially relevant studies was further analyzed for STH prevalence data.

STH infections are largely asymptomatic in nature, and it is this asymptomatic population that carries the heaviest burden of infection. Only acute cases are reported in hospitals. In this context, we included studies reporting prevalence data from community based cross-sectional studies only. Retrospective analysis and hospital based observational studies were excluded. Any study that did not report either number of participants or age group of target population or method of parasitological testing or geographical location or had discrepancy in what was reported in the abstract and full text were all excluded from the final analysis. Cross-sectional studies with full-text availability, and reporting prevalence of at least one parasite in a defined population (location, age group, number of participants) were included in the review. In case time period of the study was not specified, the date of publication was considered instead. If more than one publication was reported from the same geographical area, then most recent publication was considered for final analysis.

The data extracted from the selected publications included first author, date of publication, date of survey, state and localities where the study was carried out, sample size and age, type of parasitological testing performed, study design and percentage prevalence of each parasite. All the data was entered in an excel file and double-checked.

### Prevalence mapping

For prevalence mapping, locations where the studies were carried out were georeferenced using Google maps. A total of 49 unique locations were identified and georeferenced from where *A. lumbricoides* infections were reported. 39 unique locations were identified for *T. trichiura* and 35 locations were identified for hookworm infections.

## Results

**Fig. 1:**
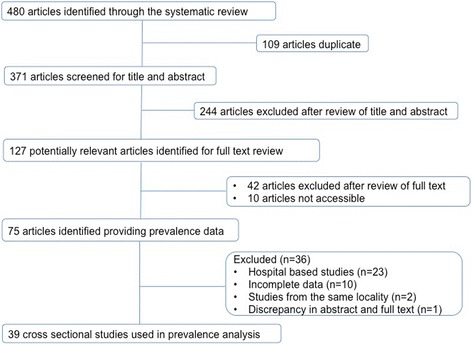
Schematic representation of the study selection process

**Fig. 2:**
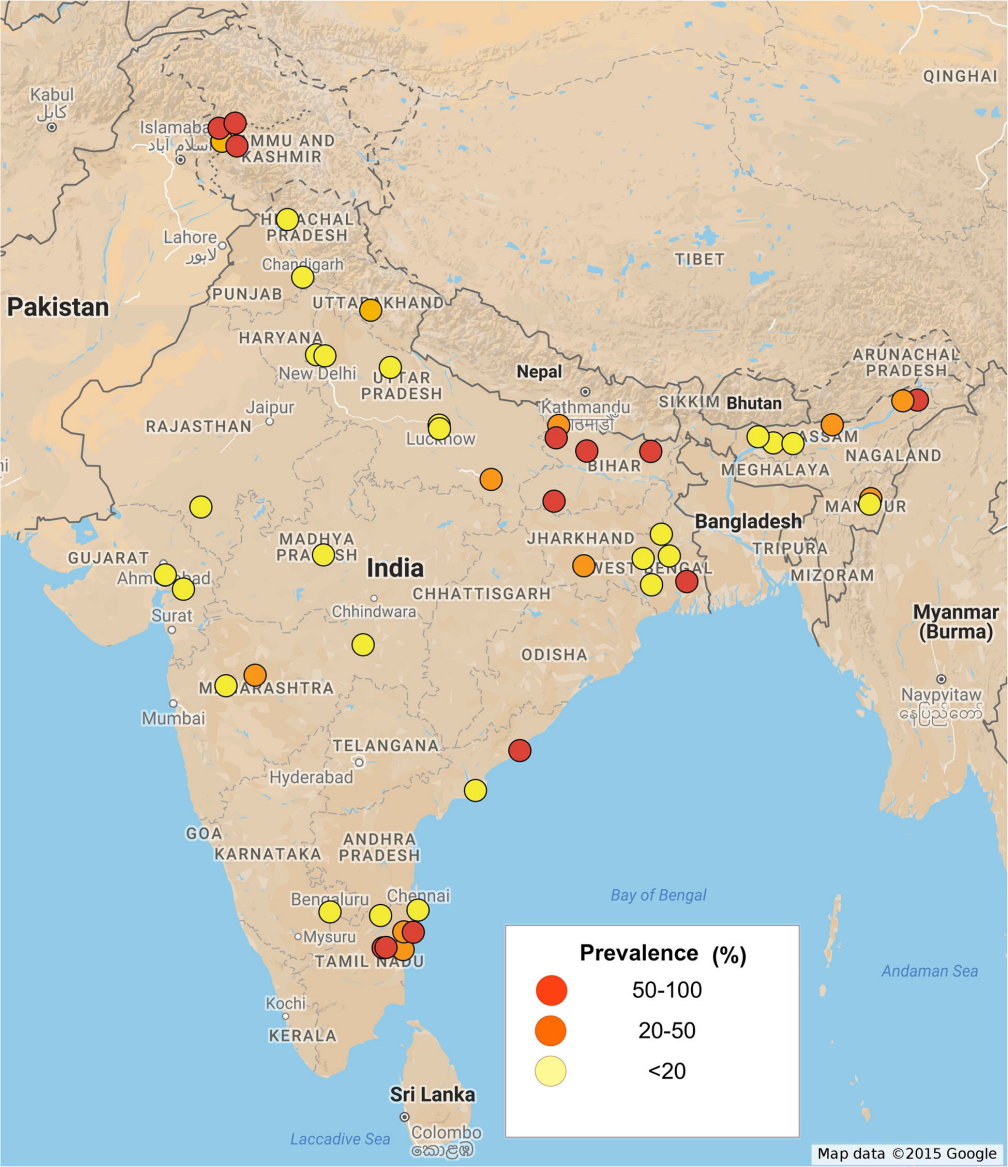
Spatial distribution of *A. lumbricoides* infections in India. Approval for reuse granted. (https://www.google.com/permissions/geoguidelines.html#general-guidelines)

**Fig. 3:**
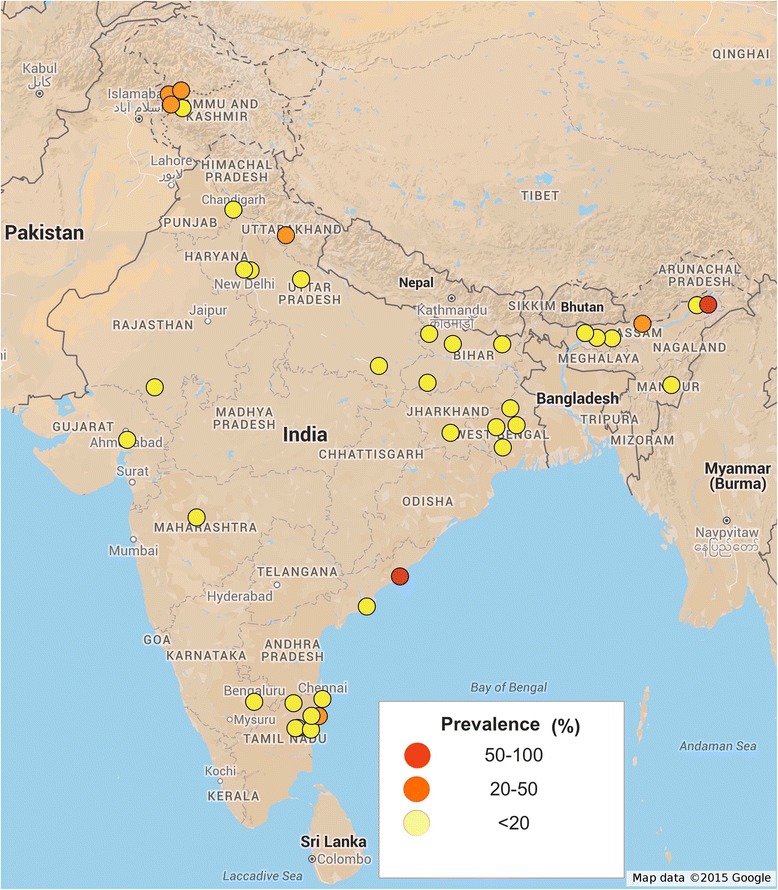
Spatial distribution of *T. trichiura* infections in India. Approval for reuse granted. (https://www.google.com/permissions/geoguidelines.html#general-guidelines)

**Fig. 4:**
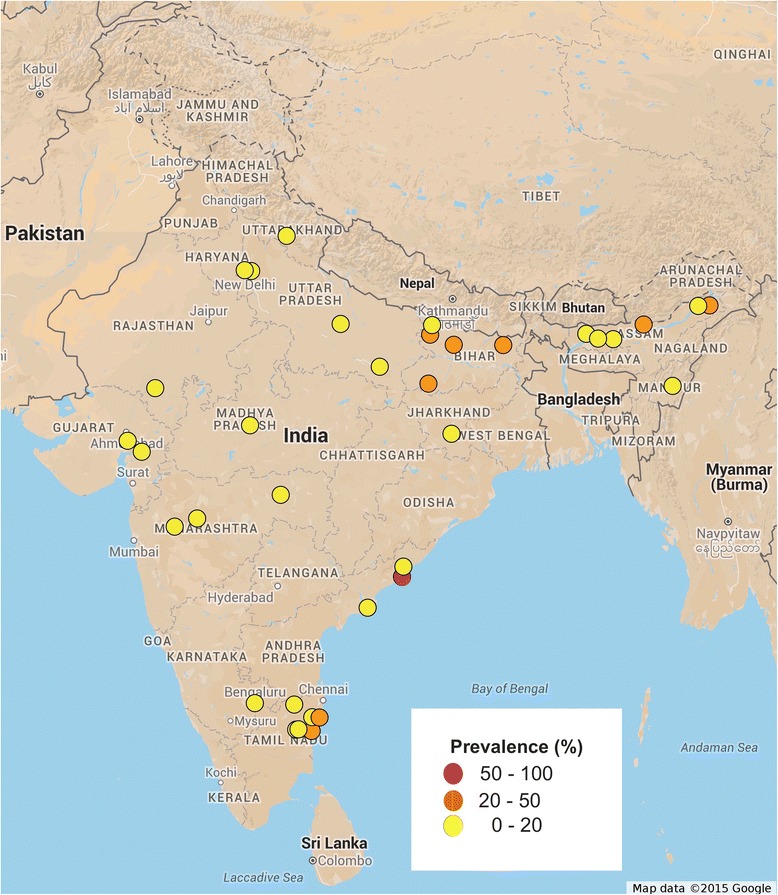
Spatial distribution of Hookworm infections in India. Approval for reuse granted. (https://www.google.com/permissions/geoguidelines.html#general-guidelines)

Initial searches identified 480 studies from PubMed and web of knowledge. After removing duplicates and irrelevant articles 127 studies were considered for full text review. Studies were excluded for not reporting prevalence data of parasites (42), lack of full text availability (10), mode of data collection (23) and incomplete data (10). Two studies were excluded as they reported data from the same geographical area; in each case only recently conducted study was considered. One study has discrepancy in what it reported in its full text and abstract. A total of 39 studies were identified that reported the prevalence of at least one STH infection among humans in India (Fig. 1). All 39 studies were cross sectional in nature with one study reporting data from pregnant females. Most of the reported data are from northern, northeastern, western and southern region of India, with a general lack of publication from northwest and southwest regions of India. Several studies (85%) reported prevalence of STH infection only in children. Smallest sample size was 25 and largest sample size was 3706. A total of 25,754 stool samples were screened for the presence of STH infection. A combination of Saline and iodine wet mount, Kato-Katz technique, salt flotation, formol-ether concentration, mini-FLOTAC and zinc sulphate concentration techniques were used for parasite detection. A total of 21 studies reported prevalence data for all three parasitic infections, 13 studies reported prevalence data for at least two parasites and 5 studies reported data only for a single parasite (Table 1). The spatial distribution of STH was spread across whole of India with a total of 49 unique locations identified and georeferenced for the prevalence of *A. lumbricoides* (Fig. 2), 39 locations for *T. trichiura* (Fig. 3), and 35 locations for hookworm (Fig. 4). Prevalence of soil-transmitted helminths varied widely with *A. lumbricoides* infection ranging from 0.6 to 91%, *T. trichiura* ranging from 0.7 to 72% and hookworm ranging from 0.02 to 52%. A higher than 50% prevalence for *A. lumbricoides* was reported from 10 different locations scattered across six states, Jammu and Kashmir, Assam, Bihar, Tamil Nadu, West Bengal and Andhra Pradesh covering nearly 30% of India’s population (Table 2) [11–49].

**Table 1:** Overview of the published reports considered for review

Characteristic	*n* (%)
No. Of studies	39 (100)
Studied Population	
Children	33 (85)
Adult	6 (15)
Parasite species reported	
All three	21 (54)
*A. lumbricoides + T. trichiura*	7 (18)
*A. lumbricoides +* Hookworm	6 (15)
*A. lumbricoides* only	4 (10)
Hookworm only	1 (3)
Stool examination method	
Direct smear only	9 (23)
Direct smear and/or salt-flotation, Zinc Sulphate flotation, Formalin-Ether concentration, mini-FLOTAC	24 (62)
Kato-katz method	6 (15)

**Table 2:** An overview of the extracted data considered for final analysis

S.No.	Author [cite ID] Year	Study Design	Study Date	Location	Sample Size	Age Group	STH (%)			Technique
							*AL*	*TT*	HW	
Kashmir										
1	Wani [11] 2008	Cross sectional	March-Nov. 2007	Kashmir valley	2256	0–15	68.3	27.92	--	Direct smear, zinc sulphate flotation, stolls egg counting technique.
2	Wani [12] 2010	Cross sectional	ND	Gurez valley	352	1–15	71.18	26.42	--	Kato-Katz thick smear technique
3	Wani [13] 2007	Cross sectional	ND	Kupwara	312	4–15	69.23	30.76	--	Direct smear and zinc sulphate flotation concentration
4	Singh [14] 2010	Cross sectional	Jan 2002 to Dec 2003	Srinagar	514	5–14	28	5	--	Iodine staining, Saline or zinc sulphate centrifugal floatation or sedimentation techniques
Delhi										
5	Ranjan [15] 2013	Cross sectional	Nov-Jan.	Civil lines zone	347	5–15	8.1	3.7	3.7	Semi quantitative katokatz technique
Chandigarh										
6	Sehgal [16] 2010	Cross sectional	Oct-Nov 2007	Urban slums	87	Pregnant women	3.4	--	--	Direct wet smear
Uttarakhand										
7	Bora [17] 2006	Cross sectional	August 2005	Pauri Garhwal,	257	9–10	28.8	1.9	5.1	Kato-katz technique
Himachal Pradesh										
8	Barda [18] 2013	Cross srctional	Jan-Apr 2012	Dharamshala	80	11–15	11.3			Direct smear, Formolether concentration, mini-FLOTAC
Uttar Pradesh										
9	Bisht [19] 2011	Cross sectional	June 2008 – Dec. 2009	Ghaziabad	335	<14	2.4	1.8	1.2	Normal saline, lugol iodine
10	Rashid [20] 2011	Cross sectional	Jan.-June 2010	Bareilly	320	5–16	9.68	1.56		Direct wet smear examination
11	Nitin [21] 2007	Cross sectional	May 2004-May 2006	Lucknow Alambagh, Mati	1071	1.5–85	A-0.6 M-2.4	--	M-0.7	Wet mount examination
12	Awasthi [22] 2008	Cross sectional	July-Sept.2007	Gyanpur	909	14.56 months mean age	41.9	2.6	6.1	Formol ether
Madhya Pradesh										
13	Tripathi [23] 2014	Cross sectional	July-Aug 2013	Kajli Kheda, Bhopal	300	6–12	4	--	0.02	Saline wet mount, Iodine wet mount
Rajasthan										
14	Choubisa [24] 2012	Cross sectional	Oct.2010-Sep. 2011	Udaipur	224	Below 60	4.46	0.44	0.89	Lugol iodine, formol ether concentration
Gujarat										
15	Shobha [25] 2013	Cross sectional	Feb-Dec 2008	Urban slum, Baroda	880	<1 to >25	2.04	--	0.11	Wet smear (saline mount) examination and iodine mount. Formalin—ether sedimentation concentration
16	Lakhani [26] 2013	Cross sectional	Jun-Oct	Piparia Village, Vadodara District	140	6–12	3.6	0.7	3.6	Saline wet mounts as well as Lugol’s Iodine wet mounts, Formol ether concentration technique
Bihar										
17	Pandey [27] 2013	Cross sectional	ND	Terai belt	404	5–65	45.79	--	8.16	Saline and iodine wet mount
18	Greenland [28] 2015	Cross sectional	Jan-Feb 2011	Araria, Aurangabad, Muzaffarpur and Gopalganj	1157	4–17	51.9	4.7	41.8	Kato-Katz smear
Jharkhand										
	Awasthi [22] 2008	Cross sectional	July-Sept. 2007	Murhu	909	14.56 months mean age	22.3	2.6	1.5	Formol ether
Assam										
19	Narain [29] 2004	Cross sectional	ND	Upper Assam	265	2-all ages	55	59	45	Formol-ether concentration method
20	Traub [30] 2004	Cross sectional	July-Sept. 2000	Phulbari Addabarie Balipara	328	0–60 year	38	43	43	Water sedimentation technique followed by zinc sulphate centrifugation, katokatz technique
21	These [31] 2009	Cross sectional	Feb-Sept 2008	Dibrugarh	1029	5 to 13	63	19	1.7	Direct and formal ether concentration methods
22	Sharma [32] 2012	Cross sectional	ND	Barpeta, Bongaigaon, Kamrup, Dibrugarh	2499	Adolescent girls, Mean 13.6 ± 1.1	10.6	6.2	3.9	Formalin-ether method
Manipur										
23	Singh [33] 2004	Cross sectional	Sept. 1998-October 2000	Urban and rural areas	1010	5–10	19.6	2.18	0.09	Wet film, iodine preparation, formol ether techniques
24	Pukhrambam [34] 2013	Cross sectional	May-Jul 2012	Urban slum (Hatta, Golapatti)	255	1–5	21.5	--	--	Saline and iodine mount method
Maharashtra										
25	Dambhare [35] 2010	Cross sectional	Oct.-Nov. 2009	Wardha (Karanji kaji village)	172	6–14	0.6	--	1.2	Iodine staining of wet mounts
26	Aher [36] 2012	Cross sectional	ND	Ahmadnagar, Loni	624	6–12 years.	1.9	--	0.9	Saline and iodine preparation
27	Avhad [37] 2012	Cross sectional	June 2011-april 2012	Aurangabad	547	9–10	22.85	10.05	1.1	Kato-katz technique
West Bengal										
28	Mukherjee [38] 2013	Cross sectional	ND	Sunderbans Bankura, birbhum Midnapore, Bardhman Kolkata	1192	10–15	11	1.8	--	Kato-katz technique
29	Sur [39] 2005	Cross sectional	May 2002-July 2003	Tiljala, Kolkata	263	2–5	53	--	--	Direct wet preparation, saline and iodine mount, formalin concentration technique
Andhra Pradesh										
30	Naish [40] 2004	Cross sectional	Dec. 1997-Dec.1998	Peda Jalaripet Vishakhapatnam	204	5–9	91	72	54	Modified formol ether sedimentation technique was used
31	Padmaja [41] 2014	Cross sectional	Nov. 2013-January 2014	Amalapuram	200	Around 8	6	7.5	4.5	Saline and iodine preparations, formal ether concentration methods.
32	Panda [42] 2012	Cross sectional	Dec 2008-Jan 2009	Kondavelagada village, Vizianagaram district	124	6–9	--	--	4.8	Saline& Iodine preparation
Puducherry										
33	Ragunathan [43] 2010	Cross sectional	March-Sept. 2006	Puducherry	1172	5–10	43.21	10.87	28.89	Formol ether sedimentation, iodine saline wet mount
Karnataka										
34	Golia [44] 2014	Cross sectional	Jun-Sep 2013	Kavalbyrasandra, Bangalore	258	6–12	8.1	5.4	1.2	Normal saline, iodine preparation and formalin ether sedimentation concentration technique.
Tamil Nadu										
35	Kattula [45] 2014	Cross sectional	Dec. 2008-Aug-2009	Vellore, thiruvanamalai	3706	6–14	1.21	0.8	6.28	Saline and iodine wet preparation, McMaster egg counting technique
36	Krishnan [46] 2013	Cross sectional	ND	Melmaruvathur	25	5–10	20	5	10	Saline and iodine preparation, salt flotation technique and zinc sulphate flotation technique
37	Fernandez [47] 2002	Cross sectional	Sept-March 2000	Chennai Kadallur	125	3–14	52.8	45.6	37.6	Saline and Lugol’s iodine zinc sulphate concentration technique
38	Dhanabal [48] 2014	Cross sectional	Jan.-Jun 2013	South Chennai	256	0–50	6.2	1.1		Saline and iodine wet mount, 10% formalin sedimentation and flotation
39	Sunish [49] 2015	Cross sectional	2001	Villupuram (Tirukoilur) (Mugaiyur)	321-T 325-M	9–10	54.83-T 52.92-M	4.67-T 6.77-M	16.51-T 8.31-M	Kato Katz cellophane thick smear technique

(*AL A. Lumbricoides, TT T. trichiura, HW Hookworm*, *ND* Not defined)

More than 50% prevalence for *T. trichiura* was reported from two different locations from the states of Assam and Andhra Pradesh and more than 50% prevalence for hookworm was reported from a single location from the state of Andhra Pradesh. Open defecation practices, lack of personal and community sanitation, lack of footwear wearing habit, poor maternal education, low literacy rate and poor socio-economic status were significant predictors of prevalence of STH infections. Jalaripet in Andhra Pradesh was a unique location from where two reports were published 7 years apart [40, 50]. A weighted average indicated more than 50% prevalence for all three parasites (91.12% for *A. lumbricoides*, 71.5% for *T. trichiura* and 50.2% for hookworm). This was the only location from where such a high prevalence was reported of all three parasites by two studies despite the time gap. The states of Uttarakhand, Uttar Pradesh, Jharkhand, Manipur, Maharashtra and Puducherry reported a prevalence higher than 20%. Less than 20% prevalence was reported from another seven states of Delhi, Himachal Pradesh, Chandigarh, Madhya Pradesh, Rajasthan, Gujarat and Karnataka

## Discussion

Soil-transmitted helminth infections continue to plague large parts of the world with India a significant contributor to the burden of disease [2]. Despite efforts to introduce usage of pit-latrines instead of open defecation, mass deworming program and improvement in water quality and sanitation, STH infections are still prevalent. A conducive climate for its growth, rapid and unplanned urbanization, social practices of open defecation and lack of community education and sanitation are some of the factors, which impedes control of infection in India. India undertook two massive deworming programme, one starting in year 2000 where a single dose of Albendazole and DEC was administered to filarial endemic regions and another in year 2015 covering 241 million children for treatment of STH infections. Although several studies have been published from different regions of India on this topic with the earliest scientific literature dating back as far as 1923 [51], the data on STH infections remains scattered. This information has the potential to inform and develop a comprehensive approach to control STH infections and target highly endemic areas with greater urgency.

The present study is a detailed effort to assess the burden of STH infections by searching past and present published literature and analyzing the prevalence of roundworm, whipworm and hookworm in one of the most endemic country in the world. The study covers 15 years of published literature on the topic of STH covering 19 out of the 29 Indian states. This study is another step in the direction of understanding prevalence and geographical distribution of STH diseases that affects nearly a one-sixth of world’s population living in India.

The most important factors affecting the survival and spread of STH infections are: the climate conditions, sanitation and socio-economic status. India has a range of climatic conditions, however except the arid and semi-arid region of Rajasthan other parts of India have largely tropical climate with high humidity and warm temperatures. Theses climatic conditions provide ideal environment for the survival of parasite eggs in moist soils, incidentally highest prevalence of STH infections was reported from such region of Tamil Nadu, Andhra Pradesh, Bihar, Assam, and West Bengal. A notable exception was Kashmir valley and Himachal Pradesh, which sees very cold and warm climates alternately, however, random urbanization without proper development of civic structure facilitates the spread of STH infections even in colder climates. Nearly 85% studies reported prevalence of STH infections in children, which form the disproportionately affected strata of society. Nearly 90% of the studies reported the prevalence of more than one parasite in same community, possibly because all the three helminths share similar climatic and socio-economic niche. Several studies reported cases of co-infection, with other protozoal parasites. Southern, northern and eastern parts of India are the most affected regions with *A. lumbricoides* the commonest parasite reported. WHO recommends annual deworming for areas with higher that 20% prevalence and biannual deworming for areas with higher than 50% prevalence (http://www.who.int/intestinal_worms/strategy/en/). Our study identifies six states with more than 20% prevalence and another six states with more than 50% prevalence rate, and in urgent need of chemotherapy. Very high prevalence was reported in Jalaripet in Andhra Pradesh implying that agriculture or farming based population may be at a greater risk due to higher exposure to contaminated soil and water at a regular basis [52, 53]. Many of these states (Delhi, Rajasthan, Tamilnadu, Assam) from which prevalence data is available, are tourist destinations. Travel based spread of vector borne diseases is well documented [54]. Similarly an exposure of naïve individuals to STH infections carries the risk of introducing STH to new locations, although tourist are a population that does not usually share lack of sanitation and poor socio-economic status and they are much less at risk of STH transmission.

Data extraction and compilation are prone to bias, to this effect we have made every effort to identify and screen published literature with broad search queries, nonetheless many relevant studies were inaccessible due to lack of full text availability. We excluded all the studies that were reported in clinical patients as STH infections are largely asymptomatic and community based cross sectional studies are better in assessing the burden of infection. Furthermore, all the studies with incomplete information were excluded which emphasizes the systematic collection of data in cross sectional studies. Additionally there was lack of publications from central, north western and southwestern region of India, with maximum studies being published from states of Tamil Nadu, Maharashtra, Assam, Uttar Pradesh and Jammu Kashmir. Odisha, Chhattisgarh and newly formed state of Telangana contribute a large section of people living below poverty line, however prevalence of this region could not be determined due to absence of any published reports. Also the review relied completely on published literature where grey literature and studies with minimal or negative results may not have been included resulting in publication bias. Another important factor that might lead to under reporting of STH infection is lack of surveillance data since STH infections are asymptomatic in nature. Only those having symptoms are expected to seek clinical intervention. A cross sectional survey from every state or union territory will accurately predict the prevalence and burden of STH infections.

WHO has recommended Kato-Katz method as the best and most reliable diagnostic tool with better efficacy, accuracy and predictive value than other techniques in resource poor settings [55]. However, only 15% studies reported the use of this method. Several states report less than 20% prevalence of STH infections. Kato-Katz technique has been shown to have reduced sensitivity in low transmission settings, which can be improved by taking more than one sample. Most of the studies relied on single stool examination, which may result in underreporting of the prevalence. Determination of prevalence and intensity of STH infection is an important tool for preventive chemotherapy and to assess the effect of ongoing deworming programs. Most of the studies have relied upon direct smear or in combination with flotation methods to determine the presence of helminth eggs. Adoption of Kato-Katz techniques with multiple stool samples will be helpful in identifying the actual prevalence of STH infections.

Despite these limitations, we were able to identify prevalence of STH infections in 19 states of India that covers nearly 84% of India’s population, clearly identifying regions of high prevalence which requires focused efforts of mass deworming to reduce parasitic load. A concomitant improvement in sanitation level will be helpful in reducing cases of reinfection. An operational focus on children by utilizing school infrastructure is generally the best approach. However targeting and educating mothers about the problems associated with helminth infections and open defecation must be emphasized by community-based programs. Systematic collection of survey reports based on WHO recommended diagnostic methods for parasitological testing should be undertaken. Such efforts will be helpful in identifying regions of high endemicity and monitor ongoing deworming programs. Geospatial mapping based on soil characteristic have been very useful in determining STH burden in other parts of the world. Such an approach could be valuable when survey data based on stool test are not available. Lastly, an overall improvement of sanitation and behavioral changes in the attitude of people will go a long way in avoiding reinfection and interrupting transmission of STH infections.

## Conclusion

The present study attempts to compile available data about the prevalence of STH infections in India. This analysis is based upon the reporting of STH prevalence data in cross sectional studies carried out in different parts of India. We have also georeferenced affected regions to identify populations in need of annual or biannual deworming to control STH infections. This study will hopefully provide a guide map for the control of STH infections by preventive chemotherapy. Furthermore, lack of studies from several parts of India requires urgent attention for the surveillance and prevalence determination of STH infection. An exhaustive knowledge of the burden of disease will be helpful in allocating resources, funding and designing survey strategies for the control and monitoring of STH infections in Indian subcontinent.

## References

[1] Savioli L, Albonico M (2004). Soil-transmitted helminthiasis. Nat Rev Microbiol.

[2] Lobo DA, Velayudhan R, Chatterjee P, Kohli H, Hotez PJ (2011). The neglected tropical diseases of India and South Asia: review of their prevalence, distribution, and control or elimination. PLoS Negl Trop Dis.

[3] http://www.who.int/neglected_diseases/preventive_chemotherapy/sth/db/?units=minimal&region=all&country=all&countries=all&year=all Accessed on 31 Oct 2015.

[4] Bethony J, Brooker S, Albonico M, Geiger SM, Loukas A (2006). Soil-transmitted helminth infections: ascariasis, trichuriasis, and hookworm. Lancet.

[5] Brooker S, Clements AC, Bundy DA (2006). Global epidemiology, ecology and control of soil-transmitted helminth infections. Adv Parasitol.

[6] Strunz EC, Addiss DG, Stocks ME, Ogden S, Utzinger J (2014). Water, sanitation, hygiene, and soil-transmitted helminth infection: a systematic review and meta-analysis. PLoS Med.

[7] Levecke B, Montresor A, Albonico M, Ame SM, Behnke JM (2014). Assessment of anthelmintic efficacy of mebendazole in school children in six countries where soil-transmitted helminths are endemic. PLoS Negl Trop Dis.

[8] Boisson S, Sosai P, Ray S, Routray P, Torondel B (2014). Promoting latrine construction and use in rural villages practicing open defecation: process evaluation in connection with a randomised controlled trial in Orissa, India. BMC Res Notes.

[9] Ziegelbauer K, Speich B, Mausezahl D, Bos R, Keiser J (2012). Effect of sanitation on soil-transmitted helminth infection: systematic review and meta-analysis. PLoS Med.

[10] Jia TW, Melville S, Utzinger J, King CH, Zhou XN (2012). Soil-transmitted helminth reinfection after drug treatment: a systematic review and meta-analysis. PLoS Negl Trop Dis.

[11] Wani SA, Ahmad F, Zargar SA, Dar PA, Dar ZA (2008). Intestinal helminths in a population of children from the Kashmir valley, India. J Helminthol.

[12] Wani SA, Ahmad F, Zargar SA, Amin A, Dar ZA (2010). Intestinal helminthiasis in children of gurez valley of Jammu and Kashmir state, India. J Glob Infect Dis.

[13] Wani SA, Ahmad F, Zargar SA, Fomda BA, Ahmad Z (2007). Helminthic infestation in children of Kupwara district: a prospective study. Indian J Med Microbiol.

[14] Singh C, Zargar SA, Masoodi I, Shoukat A, Ahmad B (2010). Predictors of intestinal parasitosis in school children of Kashmir: a prospective study. Trop Gastroenterol.

[15] Ranjan S, Passi SJ, Singh SN (2013). Prevalence and risk factors associated with the presence of Soil-Transmitted Helminths in children studying in Municipal Corporation of Delhi Schools of Delhi, India. J Parasitic Dis.

[16] Sehgal R, Reddy GV, Verweij JJ, Rao AVS (2010). Prevalence of intestinal parasitic infections among school children and pregnant women in a low socio-economic area, Chandigarh, North India. RIF.

[17] Bora D, Meena VR, Bhagat H, Dhariwal AC, Lal S (2006). Soil transmitted helminthes prevalence in school children of Pauri Garhwal District, Uttaranchal state. J Commun Dis.

[18] Barda B, Ianniello D, Salvo F, Sadutshang T, Rinaldi L (2014). “Freezing” parasites in pre-Himalayan region, Himachal Pradesh: Experience with mini-FLOTAC. Acta Trop.

[19] Bisht D, Verma AK, Bharadwaj HH (2011). Intestinal parasitic infestation among children in a semi-urban Indian population. Trop Parasitol.

[20] Rashid M, Joshi M, Joshi H, Fatemi K (2011). Prevalence of intestinal parasites among school going children in Bareilly District.

[21] Nitin S, Venkatesh V, Husain N, Masood J, Agarwal GG (2007). Overview of intestinal parasitic prevalence in rural and urban population in Lucknow, north India. J Commun Dis.

[22] Awasthi S, Verma T, Kotecha PV, Venkatesh V, Joshi V (2008). Prevalence and risk factors associated with worm infestation in pre-school children (6-23 months) in selected blocks of Uttar Pradesh and Jharkhand, India. Indian J Med Sci.

[23] Tripathi K, Nema S, Bankwar V, Dhanvijay AK (2014). Intestinal Parasitic infections and Demographic status of school children in Bhopal region of Central India. J Pharm Biol Sci.

[24] Choubisa SL, Jaroli VJ, Choubisa P, Mogra N (2012). Intestinal parasitic infection in Bhil tribe of Rajasthan, India. J Parasitic Dis.

[25] Shobha M, Bithika D, Bhavesh S (2013). The prevalence of intestinal parasitic infections in the urban slums of a city in Western India. J Infect Public Health.

[26] Lakhani JS, Rana R, Joshi S, Vasisht S (2013). Intestinal parasitic infestations among school children in Piparia Village, Vadodara District. Int J Sci Res.

[27] Pandey B, Kumar D, Verma D (2013). Epidemiological study of parasitic infestations in rural women of Terai belt of Bihar, India. Ann Biol Res.

[28] Greenland K, Dixon R, Khan SA, Gunawardena K, Kihara JH (2015). The epidemiology of soil-transmitted helminths in Bihar State, India. PLoS Negl Trop Dis.

[29] Narain K, Medhi GK, Rajguru SK, Mahanta J (2004). Cure and reinfection patterns of geohelminthic infections after treatment in communities inhabiting the tropical rainforest of Assam, India. Southeast Asian J Trop Med Public Health.

[30] Traub RJ, Robertson ID, Irwin P, Mencke N, Thompson RCA (2004). The prevalence, intensities and risk factors associated with geohelminth infection in tea-growing communities of Assam, India. Trop Med Int Health.

[31] These ME (2009). Burden of Ascariasis in schoolchildren of Assam. J Commun Dis.

[32] Sharma SK, Narain K, Devi KR, Mohapatra PK, Phukan RK (2012). Haemoglobinopathies–major associating determinants in prevalence of anaemia among adolescent girl students of Assam, India.

[33] Singh HL, Singh NB, Singh YI (2004). Helminthic infestation of the primary school-going children in Manipur. J Commun Dis.

[34] Pukhrambam R, Ranjan RK, Chaudhuri S, Mukhia S (2013). Prevalence and risk factors of soil -transmitted helminth infections among the under-five children in an urban slum of Manipur New Indian. J Pediatr.

[35] Dambhare D, Bharambe M, Garg B (2010). Intestinal parasites prevalence and related factors among school children in the rural area of central India. J Commun Dis.

[36] Aher A, Kulkarni S (2012). Prevalence of intestinal parasites in school going children in a rural community. Int J Biomed Res.

[37] Avhad S, Hiware C (2012). Soil transmitted helminthiasis among school age children in Aurangabad District, Maharashtra State, India. Prevalence.

[38] Mukherjee AK, Chowdhury P, Das K, Raj D, Karmakar S, et al. Helminth burden among school going children of southern Bengal, India: A survey report. Glob J Biol Agri Heal Sci. 2013;2(3):189–91.

[39] Sur D, Saha DR, Manna B, Rajendran K, Bhattacharya SK (2005). Periodic deworming with albendazole and its impact on growth status and diarrhoeal incidence among children in an urban slum of India. Trans R Soc Trop Med Hyg.

[40] Naish S, McCarthy J, Williams GM (2004). Prevalence, intensity and risk factors for soil-transmitted helminth infection in a South Indian fishing village. Acta Trop.

[41] Padmaja N, Swaroop PS, Nageswararao P. Prevalence of Intestinal Parasitic Infections among School Children in and around Amalapuram. J Pub Health Med Res. 2014;2(2):36-8.

[42] Panda S, Rao UD, Sankaram KR (2012). Prevalence of intestinal parasitic infections among school children in rural area of Vizianagaram. IOSR J Pharm Biol Sci.

[43] Ragunathan L, Kalivaradhan SK, Ramadass S, Nagaraj M, Ramesh K (2010). Helminthic infections in school children in Puducherry, South India. J Microbiol Immunol Infect.

[44] Golia S, Sangeetha K, Vasudha C (2012). Prevalence of parasitic infections among primary school children in bangalore. Int J Basic Appl Med Sci.

[45] Kattula D, Sarkar R, Ajjampur SSR, Minz S, Levecke B (2014). Prevalence & risk factors for soil transmitted helminth infection among school children in south India. Indian J Med Res.

[46] Krishnan A, Sekar U, Sathanantham DK. Prevalence and Pattern of Helminthic Infection among Children in a Primary School of Rural Tamil Nadu. Acad Med J India. 2013;1:40–2.

[47] Fernandez MC, Verghese S, Bhuvaneswari R, Elizabeth SJ, Mathew T (2002). A comparative study of the intestinal parasites prevalent among children living in rural and urban settings in and around Chennai. J Commun Dis.

[48] Dhanabal J, Selvadoss PP, Muthuswamy K (2014). Comparative study of the prevalence of intestinal parasites in low socioeconomic areas from South chennai, India. J Parasitol Res.

[49] Sunish I, Rajendran R, Munirathinam A, Kalimuthu M, Kumar VA (2015). Impact on prevalence of intestinal helminth infection in school children administered with seven annual rounds of diethyl carbamazine (DEC) with albendazole. Indian J Med Res.

[50] Mani GG, Rao ST, Madhavi R (1993). Estimation of hookworm intensity by anthelmintic expulsion in primary schoolchildren in south-india. Trans R Soc Trop Med Hyg.

[51] Hookworm disease in India. Br Med J. 1923;76.

[52] Clark A, Turner T, Dorothy KP, Goutham J, Kalavati C (2003). Health hazards due to pollution of waters along the coast of Visakhapatnam, east coast of India. Ecotoxicol Environ Saf.

[53] Ensink JH, Blumenthal UJ, Brooker S. Wastewater quality and the risk of intestinal nematode infection in sewage farming families in hyderabad, India. Am J Trop Med Hyg. 2008;79:561–7. http://apps.webofknowledge.com/full_record.do?product=WOS&search_mode=GeneralSearch&qid=2&SID=4Ews1NjaqvMUbdx4wqN&page=1&doc=1. PMC2665018 18840745

[54] Nunes MR, Palacios G, Faria NR, Sousa EC, Pantoja JA (2014). Air travel is associated with intracontinental spread of dengue virus serotypes 1-3 in Brazil. PLoS Negl Trop Dis.

[55] Nikolay B, Brooker SJ, Pullan RL (2014). Sensitivity of diagnostic tests for human soil-transmitted helminth infections: a meta-analysis in the absence of a true gold standard. Int J Parasitol.

